# Cost-effectiveness of multimodal intervention for the prevention of dementia in Japan

**DOI:** 10.1016/j.tjpad.2025.100460

**Published:** 2026-01-01

**Authors:** Naoki Takashi, Shosuke Ohtera, Yujiro Kuroda, Hidenori Arai, Takashi Sakurai, J-MINT investigators

**Affiliations:** a Department of Health Economics, Center for Gerontology and Social Science, Research Institute, National Center for Geriatrics and Gerontology, 7-430 Morioka-cho, Obu City, Aichi 474-8511, Japan; b Department of Prevention and Care Science, Center for Development of Advanced Medicine for Dementia, Research Institute, National Center for Geriatrics and Gerontology, 7-430 Morioka-cho, Obu City 474-8511, Aichi, Japan; c National Center for Geriatrics and Gerontology, 7-430 Morioka-cho, Obu City, Aichi 474-8511, Japan; d Research Institute, National Center for Geriatrics and Gerontology, 7-430 Morioka-cho, Obu City, Aichi 474-8511, Japan

**Keywords:** Cost-effectiveness, Dementia prevention, Multimodal intervention, Health economic simulation, Japan

## Abstract

**Background:**

This analysis evaluated the potential cost-effectiveness of the Japan-Multimodal Intervention Trial for the prevention of dementia (J-MINT), targeting older adults with mild cognitive impairment (MCI) from a societal perspective.

**Methods:**

Using a time-dependent cohort state-transition model, we estimated the long-term economic impact of J-MINT. Costs included medical, long-term, and informal care. Incremental cost-effectiveness ratios (ICERs) were calculated based on simulated costs and quality-adjusted life years (QALYs).

**Results:**

The base-case analysis indicated that the J-MINT was dominant, demonstrating cost saving and more effective compared to usual care. Over 35 years, J-MINT was projected to achieve cost savings of JPY 452,826 per person and a gain of 0.08 QALYs. Deterministic and probabilistic sensitivity analyses confirmed the robustness of these findings. Scenario analysis suggested that targeting APOE ε4 carriers or individuals with high adherence to exercise yielded even greater benefits.

**Conclusion:**

J-MINT demonstrates cost-effectiveness by reducing overall care costs while improving QALYs in individuals with MCI.

## 1 Introduction

Dementia is a major public health concern worldwide. In 2019, approximately 57 million individuals worldwide were affected, with projections suggesting that this number may reach 153 million by 2050 [1]. Ranked as the seventh leading cause of death worldwide, dementia imposes SUBSTANTIAL burdens not only on persons living with dementia but also on families, carers, and the healthcare systems [2]. These burdens range from increased healthcare resource use to significant informal care demands. Notably, in Japan, an aging society with one of the highest proportions of older adults worldwide, cultural norms place a strong emphasis on family-based care, potentially exacerbating the informal caring load and influencing the overall cost structure of dementia care [[3], [4], [5]].

Dementia-related cost reached US$1.3 trillion globally in 2019, with informal care accounting for nearly half [6]. In Japan, this burden may be greater due to family caregiving traditions and limited long-term care services. Cultural and demographic factors, such as lifestyle, genetic (e.g., APOE ε4), and social environment, can influence dementia progression and the impact of preventive measures [[7], [8], [9]]. Given Japan’s aging population, evaluating the cost-effectiveness of prevention strategies is increasingly important for maintaining sustainable care systems [10].

Identifying effective approaches to reducing dementia incidence offers dual benefits: supporting better health outcomes for older adults and promoting the long-term viability of healthcare systems. Previous UK-based analyses suggested that preventive measures can lower medical costs by approximately 23 % [11]. These findings underscore the importance of interventions that balance clinical effectiveness and economic feasibility, particularly in societies facing significant resource allocation challenges.

Recent findings from the 2024 Lancet Commission on Dementia reinforce the idea that addressing key risk factors can prevent or delay up to half of all dementia cases [12]. Beyond previously recognized factors, such as limited education, hearing loss, hypertension, smoking, obesity, depression, physical inactivity, diabetes, social isolation, traumatic brain injury, air pollution, and excessive alcohol consumption, the Commission's latest report highlights untreated vision loss and high LDL cholesterol as additional risks. Given the global aging trend, sustained life-course interventions can play a critical role in reducing multiple modifiable dementia risk factors.

These insights have prompted investigations into multidomain interventions that simultaneously address multiple risk factors. For example, the Finnish Geriatric Intervention Study to Prevent Cognitive Impairment and Disability (FINGER), for example, demonstrated that combining dietary guidance, exercise, cognitive training, and vascular risk management could slow cognitive decline in older adults at risk of dementia [13]. Furthermore, cost-effectiveness modeling of the FINGER approach suggested long-term economic value, with cost savings and QALY gains relative to usual care [14]. In addition, multidomain interventions have been suggested to be cost-effective even within a relatively short time horizon of three years, in terms of reducing cognitive decline and dementia risk [15].

However, it is unclear whether these findings apply to Asian populations, as prior studies predominantly focus on Western cohorts. While some cost-effectiveness analyses have been conducted in Asian populations, they were based on short time horizons—such as two years post-intervention—limiting their generalizability to long-term dementia prevention [16]. Little is known about the long-term economic impact of multidomain interventions in older Japanese adults, who may differ in genetics, cultural context, and caring patterns. This knowledge gap is especially critical among individuals classified as having mild cognitive impairment (MCI), a population that is often excluded or underrepresented in cost-effectiveness analyses. Although the Japan-Multimodal Intervention Trial for the Prevention of Dementia (J-MINT) has investigated a multidomain program in Japanese older adults with MCI, comprehensive long-term economic evaluations are still lacking [17,18].

Addressing this gap is essential for guiding clinical practice and informing policymakers, healthcare planners, and stakeholders responsible for resource allocation. Given the intensifying strain on Japan's healthcare and caring infrastructure, robust cost-effectiveness data can support evidence-based decision-making, helping determine whether scaling up such interventions would be a prudent long-term investment. By providing insights into clinical and economic outcomes, this study offers valuable reference points for countries facing similar demographic transitions and resource constraints.

This study aimed to evaluate the cost-effectiveness of the J-MINT program compared with usual care in older Japanese adults with MCI by estimating differences in lifetime costs and quality-adjusted life years from a societal perspective using a time-dependent cohort state-transition model. The findings are intended to strengthen the evidence base for informing future dementia prevention policies and strategies.

## 2 Methods

### 2.1 J-MINT project

The J-MINT study was an 18-month, multicenter, randomized controlled trial conducted at five medical institutions in Japan [17]. The participants were individuals aged 65–85 with mild cognitive impairment recruited from hospitals, memory clinics, and community-based cohorts. Among the participants, the number of APOE ε4 carriers was 70 out of 212 (33 %) in the intervention group and 54 out of 214 (25 %) in the control group. Hypertension was observed in 100 (47 %) and 100 (46 %) patients in the intervention and control groups, respectively; hyperlipidemia was noted in 80 (37 %) and 77 (35 %) patients, respectively; and diabetes was reported in 32 (15 %) and 36 (17 %) patients, respectively.

In addition to the primary analysis, the RCT included subgroup analyses targeting individuals expected to benefit from the intervention, such as APOE ε4 carriers and those with a group-based exercise session attendance rate of 70 % or higher (high adherence).

The 18-month J-MINT program includes four key components: vascular risk management, structured exercise, nutritional counseling, and cognitive training. In the management of vascular risk factors, participants received treatment for diabetes, hypertension, and dyslipidemia in accordance with the Japanese clinical practice guidelines. For group-based physical exercise, the participants were encouraged to attend 78 group exercise sessions, each lasting 90 min, once a week. The sessions included stretching, strength training, aerobic exercises, dual-task training, and group meetings. During the group meetings, health-related information was provided to promote healthy behaviors, and participants were encouraged to discuss their physical, social, and cognitive activities. Nutritional counseling was conducted on an individual basis. The participants received three face-to-face counseling sessions (each lasting 60 min) and 12 telephone counseling sessions over the intervention period. The first six months focused on improving lifestyle and dietary habits. In comparison, the subsequent 7–18 months included guidance on dietary intake necessary for improving cognitive and physical functions, instructions on chewing and swallowing functions, and oral care. For cognitive training, participants engaged in self-directed cognitive exercises using a customized version of "Brain HQ" tailored for the J-MINT study.

On the other hand, participants in the control group received the management of vascular risk factors along with general health-related information in written form every two months.

The trial was approved by the Ethics Committee of Human Research at the National Center for Geriatrics and Gerontology (No. 1791).

### 2.2 Study design

To evaluate the cost-effectiveness of the J-MINT program compared to usual care, this study used quality-adjusted life years (QALYs) as a measure of effectiveness and applied a time-dependent cSTM for analysis from a societal perspective. This study aimed to estimate the impact of an 18-month J-MINT program for adults aged 65 years and above with MCI on lifetime costs and quality of life-related to dementia onset. Observations of healthcare and long-term care service use and quality of life during the trial period did not capture the potential lifetime benefits beyond the trial duration [19]. Therefore, a cSTM was chosen to simulate lifetime health-economic effects. Furthermore, since dementia onset risk, mortality, and quality of life are assumed to change with aging, a time-dependent cSTM was employed to account for these time-dependent effects [20]. Usual care was defined as the management of vascular risk factors combined with the provision of general health-related information every two months, as implemented in the control group of the RCT.

This manuscript follows the Consolidated Health Economic Evaluation Reporting Standards (CHEERS) guidelines to ensure methodological rigor, transparency, and consistency in reporting health-related economic evaluations [21].

**Fig. 1:**
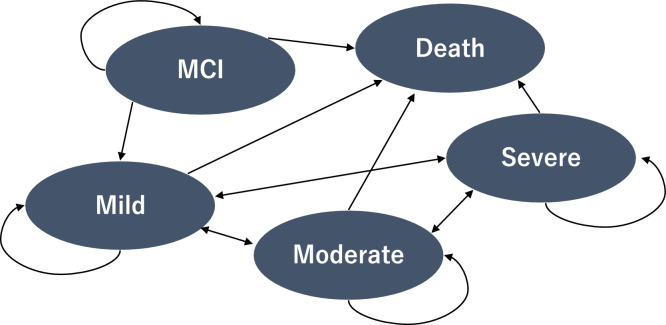
The basic structure of the five-state time-dependent cSTM.

The time-dependent cSTM defined the following five states: MCI, mild dementia, moderate dementia, severe dementia, and death. In this model, all transitions from MCI to dementia are defined as transitions to mild dementia. Fig. 1 visually represents these five health states and the permitted transitions between them, illustrating how individuals move through different stages of cognitive decline over time in the model. The model assumes that all individuals with incident dementia transition first to mild dementia rather than directly to moderate dementia, based on the low likelihood of direct progression to moderate severity at the onset of dementia. This assumption is consistent with the results of a previous study [14]. The starting age of the model was set at 65 years, reflecting the eligibility criteria of the J-MINT program [17]. Each cycle was defined as one year, and the model simulated the incidence and natural progression of dementia over 35 cycles up to the age of 100 years. Dementia-related costs and QALYs over 35 years from age 65 were estimated, and the incremental cost-effectiveness ratio (ICER) was calculated.

Cycle correction was performed using Simpson's 1/3 rule [22]. The discount rate was set at 2 % per Japanese guidelines [23]. All costs are expressed in 2023 Japanese yen (JPY), converted to US dollars, using the purchasing power parity (PPP) exchange rate.

Programming was conducted using R, and the model was constructed based on the code developed by Alarid-Escudero et al [20,24].

### 2.3 Model input

#### 2.3.1 Incidence of dementia

The transition probabilities from MCI to mild dementia were based on age-specific dementia incidence rates (60 s, 70 s, and 80+ years) reported in a previous Japanese study [25].

#### 2.3.2 Disease progression

The transition probabilities between the states were based on the data used in the cost-effectiveness analysis conducted in the FINGER study. Despite performing literature reviews and manual searches, obtaining corresponding data within Japan has proved challenging. Therefore, the transition probabilities from the FINGER study, which employed a similar model, were referenced. These probabilities were derived from data collected by SveDem, the Swedish Dementia Registry. SveDem, established in 2007, conducts annual follow-up studies on over 100,000 individuals with dementia.

#### 2.3.3 Mortality

The age-specific mortality rate of individuals in the MCI state was assumed to be equivalent to that of the general population. Age-specific mortality data for the general population were obtained from the 2020 life tables published by the Japanese government. Mortality rates for mild, moderate, and severe dementia were estimated by applying the hazard ratios reported in a previous Japanese study [26] to age-specific mortality rates.

#### 2.3.4 Intervention effect

The effect of the J-MINT program on dementia onset risk was challenging to analyze directly using RCT data, owing to the short follow-up period and low incidence of dementia cases. To address this, an indirect risk assessment was performed using the Hisayama score at baseline and 18 months post-intervention for both the intervention and control groups. The Hisayama score is a validated tool designed to estimate the 10-year risk of dementia onset in Japanese individuals aged 65 and older. It accounts for multiple risk factors, including age, sex, educational history, low BMI, hypertension, diabetes, history of stroke, smoking status, and physical activity. A higher score indicated a greater predicted risk of developing dementia within 10 years [27].

This study estimated relative risk reduction between the intervention and control groups using changes in Hisayama scores. Analysis of the J-MINT study revealed that during the 18-month follow-up period, the Hisayama score increased by 0.78 points in the control group but only by 0.56 points in the intervention group. This suggests that the increase in the Hisayama score was suppressed by 0.22 points in the intervention group compared to the control group. Consequently, the average Hisayama score in the control group (5.29 points) was projected to decrease to 5.07 points with the implementation of the J-MINT program.

By substituting the total score in the equation $P=1-0.8254^{\text{exp}(total\;score\;\times 0.302-1.543)}$, the 10-year dementia risk was calculated as 0.183 for the control group (Pc) and 0.173 for the intervention group (Pi). This corresponded to a relative risk reduction of 5.8 % and a relative risk of 0.942. The base-case analysis assumed that the intervention effect of the J-MINT program would persist over the participants’ lifetimes following the end of the program, in line with assumptions adopted in a previous study [14]. This relative risk was applied across all age groups from 65 to 100 years to estimate the lifetime impact of the J-MINT program.

#### 2.3.5 Costs

This study considered medical care costs, public long-term care costs, and informal care costs. Our estimates were derived from a previous study [3], which calculated annual per capita costs based on dementia severity using publicly available data sources, including academic articles, research grant reports from the Ministry of Health, Labour and Welfare, and government statistical surveys in Japan.

Medical care costs consisted of drug and non-drug components. Drug costs were estimated based on the annual total cost of four Alzheimer’s disease medications (donepezil, rivastigmine, galantamine, and memantine) using prescription volumes and official drug prices. Non-drug costs were derived from a physician survey that reported average annual dementia-specific direct costs by disease severity based on actual clinical practice. Public long-term care costs accounted for the distribution of care need levels by dementia severity and included both facility-based and home-based services. In Japan’s long-term care insurance system, service use is capped by benefit limits tied to care need levels [28]. Informal care costs were estimated based on caregiving time for activities of daily living (ADL) and instrumental ADL, stratified by care need levels, and valued using age- and sex-specific wage data [3].

The cost of implementing the program was calculated based on detailed information from the actual RCT (as shown in Supplemental Table 1), including development costs, types of professionals involved, and the frequency and number of sessions, resulting in an estimated per capita cost for the 18-month intervention.

#### 2.3.6 QALYs

QALYs were calculated using health-related quality of life (HRQoL) estimates for each health state, stratified by age group (65–69 years, 70–79 years, 80–89 years, 90–100 years) based on a previous study [29]. The EQ-5D-5 L scale was used as the measurement tool in this study. For the MCI state, it was assumed that HRQoL utility values were equivalent to the population norm for the general Japanese population. The disutility (reduction in utility value) associated with dementia onset was set at 0.222 based on the previous research [29]. Additionally, the utility values after dementia onset remained constant regardless of disease severity [30].

#### 2.3.7 Sensitivity analysis

Deterministic and probabilistic sensitivity analyses were conducted to account for uncertainties in the parameters and assumptions. Scenario analyses were performed to evaluate the heterogeneity in outcomes across subgroups.

### 2.4 Deterministic sensitivity analysis

#### 2.4.1 Model length

Shorter modeling periods of 5, 10, and 20 years were considered in this study.

#### 2.4.2 Incidence of dementia

Our analysis was conducted under the assumption that the target population of the J-MINT program consisted of the general older adult population at lower risk of dementia onset, specifically community-dwelling individuals without cognitive impairment.

#### 2.4.3 Discount rate

Discount rates of 1 % and 5 % were considered.

#### 2.4.4 Intervention costs

The intervention costs varied and we analyzed scenarios assuming extended intervention durations beyond the base-case 18 month program. Specifically, we considered durations of approximately 2 years (+50 %), 3 years (+100 %), and 4.5 years (+200 %). These scenarios were informed by previous systematic reviews [31,32], which reported similar multidomain interventions, including physical activity, nutritional counseling, and cognitive training or stimulation, with durations of up to 3 years. Notably, the 4.5 year scenario exceeds the maximum intervention duration reported in the literature and was included to explore the potential impact and cost implications of prolonged implementation. In all scenarios, intervention costs were modeled as a one-time expense, regardless of whether participants remained in the MCI state.

#### 2.4.5 Duration of intervention effect

The base-case analysis assumed that the intervention effect would persist throughout participants’ lifetimes after program completion. Although this assumption is consistent with that adopted in a previous study [14], it may lead to an overestimation of the cost effectiveness of the J-MINT program. To address the uncertainty surrounding this assumption, additional analyses were conducted assuming that the intervention effect would last for 3, 5, 10, 20, or 30 years; or that the effect would decline by 15 % annually, either over the participants’ lifetimes or over a 10-year period, after which the effect was assumed to cease entirely. The latter assumption was informed by the observation that approximately 22 % of participants dropped out over 18 months in the J-MINT trial [17]. The choice of a 10 year duration was made based on the predictive horizon of the Hisayama risk score, which estimates dementia onset risk over a 10 year period [27].

#### 2.4.6 Probabilistic sensitivity analysis

Probabilistic sensitivity analysis was conducted to simultaneously account for multiple uncertainties and evaluate the robustness of the base case results [[22], [23], [24]]. Appropriate probability distributions were assigned to each parameter, and 10,000 parameter sets were sampled from these distributions. The expected costs and effects of the intervention (J-MINT program) and comparator (usual care) were then simulated. The values required to set the distributions for transitional probabilities and costs were difficult to obtain from the previously mentioned literature. Therefore, they were extracted from the following studies [33,34]. Based on the results, a cost-effectiveness scatterplot and 95 % confidence ellipse were generated.

#### 2.4.7 Scenario analysis

Scenario analyses were conducted assuming that the program was applied exclusively to the subgroups that demonstrated particularly strong effects in preventing cognitive decline in the RCT. These subgroups included APOE ε4 carriers and individuals with a high adherence rate (≥70 %) to group-based exercise sessions.

## 3 Results

The parameters and probability distributions used in the analysis are summarized in Table 1.

**Table 1:** Model input.

Parameter			Value	Source	Distribution
Incidence rate for dementia onset (per 1000 person-years)				Meguro K et al., 2007	
	Aged 65–69		23.08		rgamma (143.14, 0.0004)
	Aged 70–79		55.76		
	Aged 80 and more		100.86		
Transition probabilities				Wimo A et al., 2022	
	Mild to Mild		0.75		rgamma (649.73, 0.002)
	Mild to Moderate		0.25		rgamma (659.17, 0.0004)
	Mild to Severe		0.00		0
	Moderate to Mild		0.09		rgamma (81.84, 0.001),
	Moderate to Moderate		0.83		rgamma (2443.75, 0.0007)
	Moderate to Severe		0.09		rgamma (25.93, 0.004)
	Severe to Mild		0.03		rgamma (393.64, 0.00008)
	Severe to Moderate		0.13		rgamma (387.37, 0.0004)
	Severe to Severe		0.85		rgamma (796.14, 0.001)
Hazard ratios for mortality				Takata Y et al., 2014	
	Mild		1.42		rlnorm (log(1.42), 0.37)
	Moderate		2.59		rlnorm (log(2.59), 0.30)
	Severe		5.23		rlnorm (log(5.23), 0.15),
Relative risk for intervention effect			0.942	Calculated using J-MINT trial data	rlnorm (log(0.942), 0.03)
Healthcare costs, including drug costs (annual)				Ikeda S et al., 2021	
	Mild		JPY 317,029		N/A
	Moderate		JPY 460,909		N/A
	Severe		JPY 601,117		N/A
Public long-term care costs (annual)					
	Mild		JPY 919,814		N/A
	Moderate		JPY 1790,152		N/A
	Severe		JPY 2733,045		N/A
Informal care costs (annual)			JPY 1877,077		N/A
Total costs					
	Mild		JPY 3113,920		rgamma (13,301.09, 234.11),
	Moderate		JPY 4128,138		rgamma (23,376.58, 176.59)
	Severe		JPY 5211,239		rgamma (37,252.42, 139.89)
Intervention costs (18 months)				Calculated using J-MINT trial data	
	J-MINT program		JPY 520,962		N/A
	Standard of care		JPY 432		N/A
Utility				Shiroiwa T et al., 2021.	
	MCI				
		65–69	0.93		rbeta (2935.71, 227.77)
		70–79	0.88		rbeta (2626.76, 348.05),
		80–89	0.80		rbeta (1949.98, 493.60),
		90–100	0.74		rbeta (2168.012, 777.66),
	Dementia (including mild, moderate, and severe)				
		65–69	0.71		rbeta (6939.60, 2889.86)
		70–79	0.66		rbeta (4265.74, 2187.72),
		80–89	0.58		rbeta (1382.55, 183.19),
		90–100	0.51		rbeta (1946.70, 1840.65)

### 3.1 Results of the base case analysis

The results of the base-case analysis indicated that the ICER was dominant, suggesting that the J-MINT program has the potential to be a cost-effective alternative to usual care (Table 2). Specifically, over 35 years, the J-MINT program is projected to achieve cost savings of JPY 452,826 per person and a gain of 0.08 QALYs compared to usual care.

**Table 2:** Base case results.

	Incremental Costs	Incremental QALY	ICER		Expected Cost_ SOC	Expected Cost _JMINT	QALY_SOC	QALY_JMINT
Base case	−452,826 (−4192.06)	0.08	Dominant		JPY 19,128,094 (USD 177,079.19)	JPY 18,675,268 (USD 172,887.13)	12.45	12.54

### 3.2 Results of sensitivity analyses

**Fig. 2:**
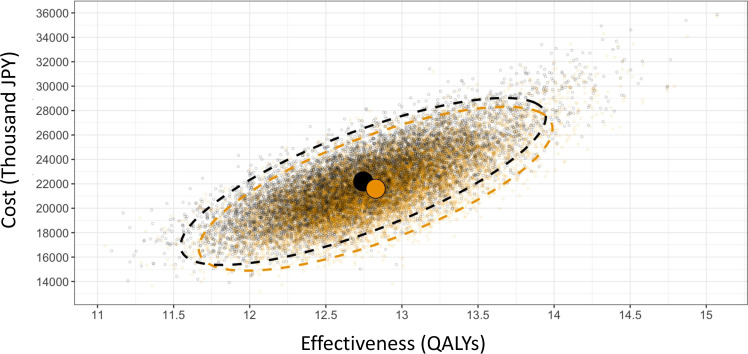
Probabilistic sensitivity analysis results.

A series of sensitivity analyses consistently supported the results of the base case analysis, indicating that the J-MINT program was more cost-effective than usual care (Tables 3,4, Fig. 2).

**Table 3:** One-way sensitivity analyses results.

	Incremental Costs (JPY)	Incremental QALY	ICER		Expected Cost_SOC (JPY)	Expected Cost_JMINT (JPY)	QALY_SOC	QALY_JMINT
Model length 20 years	−368,275	0.05	Dominant		14,037,883	13,669,608	11.24	11.29
Model length 10 years	21,960	0.01	1606,600		4001,238	4023,198	7.06	7.07
Model length 5 years	178,415	0.00	55,945,293		862,585	1041,000	3.96	3.96
Low risk: general population	47,709	0.02	1966,629		3438,917	3486,626	14.45	14.48
Discount rate 1 %	−544,866	0.1	Dominant		22,308,452	21,763,586	13.72	13.82
Discount rate 5 %	−249,682	0.05	Dominant		12,451,934	12,202,252	9.61	9.66
Intervention cost +50 %	−339,805	0.08	Dominant		19,128,094	18,788,289	12.45	12.54
Intervention cost +100 %	−226,784	0.08	Dominant		19,128,094	18,901,310	12.45	12.54
Intervention cost +200 %	−741	0.08	Dominant		19,128,094	19,127,353	12.45	12.54
Sustained effectiveness 30 years	−451,767	0.08	Dominant		19,128,094	18,676,327	12.45	12.54
Sustained effectiveness 20 years	−418,070	0.08	Dominant		19,128,094	18,710,024	12.45	12.53
Sustained effectiveness 10 years	−218,043	0.05	Dominant		19,128,094	18,910,051	12.45	12.51
Sustained effectiveness 5 years	12,156	0.02	510,627		19,128,094	19,140,250	12.45	12.48
Sustained effectiveness 3 years	109,028	0.01	8612,037		19,128,094	19,237,122	12.45	12.47
Decline by 15 % annually over the participants’ lifetimes	−31,163	0.03	Dominant		19,128,094	19,096,931	12.45	12.48
Decline by 15 % annually over a 10-year period	−7428	0.03	Dominant		19,128,094	19,120,666	12.45	12.48

**Table 4:** Probabilistic sensitivity analysis results.

	Incremental Costs	Incremental QALY	ICER		Expected Cost_ SOC	Expected Cost _JMINT	QALY_SOC	QALY_JMINT
Probability sensitive analysis	−596,948 (−5526.27)	0.08	Dominant		JPY 22,194,090 (USD 205,462.78)	JPY 21,597,142 (USD 199,936.51)	12.75	12.83

### 3.3 Deterministic sensitivity analysis

The results of deterministic sensitivity analysis showed that model length was a key factor influencing outcome uncertainty (Table 3). When the model length was reduced to 10 or 5 years, the QALYs for the J-MINT program were slightly higher than those for usual care, although the expected costs were also higher than usual care. The ICERs were calculated as 1606,600 JPY and 55,945,293 JPY, respectively. Similarly, the risk of dementia onset has also been identified as an important factor. When the program was applied to the general population, the QALYs for the J-MINT program were only modestly higher than those for usual care (usual care: 14.45, J-MINT: 14.48), whereas the expected costs for the J-MINT program exceeded those for usual care by 47,709 JPY, resulting in an ICER of 1966,629 JPY.

In contrast, the duration of the intervention effect had little impact on the uncertainty of the results. Even when the effect duration varied between 10 and 30 years, the ICER remained dominant, indicating that the program consistently maintained cost-effectiveness under these assumptions.

### 3.4 Probabilistic sensitivity analysis

The results of the probabilistic sensitivity analysis support the findings of the base-case analysis (Table 4). In the cost-effectiveness scatterplot (Fig. 2), 10,000 simulations are plotted as points on the graph. Orange points, representing the J-MINT program, tend to show higher effectiveness and lower costs than black points, representing usual care.

### 3.5 Scenario sensitivity analysis

The results of the scenario analysis indicated that applying the program exclusively to APOE ε4 carriers or individuals with high adherence to exercise sessions could achieve greater cost savings and additional QALYs compared to the base case (Table 5). Across various sensitivity analyses, J-MINT consistently remained cost-effective, reinforcing the robustness of our findings.

**Table 5:** Scenario analyses results.

	Incremental Costs	Incremental QALY	ICER		Expected Cost_ SOC	Expected Cost _JMINT	QALY_SOC	QALY_JMINT
APOE ε4	−1684,387 (−15,593.29)	0.24	Dominant		JPY 19,128,094 (USD 177,079.19)	JPY 17,443,707 (USD 161,485.90)	12.45	12.69
High adherence to exercise sessions	−535,873 (−4960.87)	0.1	Dominant		19,128,094 (USD 177,079.19)	18,592,221 (USD 172,118.32)	12.45	12.55

## 4 Discussion

This study confirms that J-MINT program is a cost-effective strategy for dementia prevention in older adults with mild cognitive impairment (MCI), reducing care-related costs while improving QALYs. While previous economic evaluations have largely focused on individuals at-risk or dementia patients, our findings indicate that multimodal interventions can be cost-effective even at the MCI stage. Given that MCI is a critical window for dementia prevention, incorporating cost-effective early interventions into public health strategies could have long-term benefits in reducing dementia prevalence and associated costs.

As the number of individuals diagnosed with MCI is expected to increase in Japan [35], effective dissemination of such programs, driven in part by lifestyle improvements like increased physical activity, could yield substantial societal benefits.

Some multimodal interventions have primarily targeted populations considered to be at risk of dementia due to lifestyle factors rather than cognitive decline [36]. For example, a previous cost-effectiveness analysis of the FINGER study demonstrated that multimodal interventions were cost-effective for population at risk of dementia defined based on ‘Cardiovascular Risk factors, Aging and Dementia’ (CAIDE) Risk [14]. The findings of this study further reveal that multimodal interventions can also achieve favorable cost-effectiveness in populations with MCI.

The findings from the deterministic sensitivity analysis suggest that the results of the base case analysis are sensitive to dementia incidence rates. Specifically, when dementia incidence risk was reduced in the sensitivity analysis, the incremental cost-effectiveness ratio (ICER) increased. This trend indicates that the uncertainty in dementia incidence contributes to the overall uncertainty in our results. Recent studies have reported a declining trend in the incidence of dementia in high-income countries, and a similar trend may occur in Japan [37]. As the incidence rate data used in this study were based on estimates from approximately 20 years ago, future research should incorporate more recent incidence data.

The base-case analysis in this study assumed that the intervention effect would persist throughout participants’ lifetimes following program completion. Although this assumption aligns with that adopted in a previous study [14], its validity remains debatable [38]. Therefore, sensitivity analyses were conducted assuming intervention durations of 3, 5, 10, 20, and 30 years, as well as scenarios in which the intervention effect declined by 15 % annually, either over the participants’ lifetimes or over a 10 year period, after which the effect was assumed to cease. Favorable cost effectiveness was observed in all scenarios except when the duration was limited to 3 years. When the assumed duration of effect was 5 years, the ICER was approximately JPY 510,000 per QALY gained, which is well below the commonly accepted threshold (JPY 5000,000 per QALY), and considered to be within an acceptable range.

However, conclusive evidence from long-term randomized trials demonstrating that lifestyle interventions reduce cognitive decline or the incidence of dementia is still lacking. Furthermore, the extent to which lifestyle improvements achieved through such programs can be maintained over time remains uncertain, making it difficult to determine the duration of the intervention effect accurately. In a previous study with up to 12 years of follow-up after a 3 year multi-domain intervention, a trend toward reduced cognitive decline was observed among participants with lower baseline MMSE scores and those carrying the ApoE ε4 allele, although the results were not statistically significant [39].

The absence of conclusive long-term evidence presents a challenge for policymakers in determining an appropriate course of action. Possible options include delaying implementation until more robust evidence becomes available, proceeding with wide-scale implementation under the assumption that the current evidence and assumptions are sufficiently reliable, or investing in additional research to strengthen the evidence base.

As highlighted in previous studies [39], evaluating the long-term effects of multidomain interventions is inherently difficult, especially among individuals at higher risk of dementia such as those with low MMSE scores at baseline due to challenges in maintaining long-term follow-up. A potential solution is to use administrative data, which enables the long-term tracking of diagnostic and healthcare outcomes with minimal burden and reduced attrition bias [40,41]. Linking trial data with such data sources could provide a pragmatic approach to long-term evaluation. When considering the scaling up of multi-domain interventions such as the J-MINT program at the municipal level, local decisionmakers may wish to invest in the development of systems that facilitate administrative data linkage to support extended follow-up and evaluation.

Furthermore, multi-domain interventions have been reported to contribute to the prevention of chronic diseases [42]. Although this study focused solely on dementia-related outcomes, if such interventions are also effective at preventing other age-related diseases, the current analysis may underestimate overall benefits. These potential spillover effects may also inform policymakers’ decisions regarding large-scale implementation.

While the J-MINT program demonstrated favorable cost-effectiveness within the controlled environment of a randomized trial, several implementation challenges must be addressed before large-scale adoption. Real-world feasibility may be influenced by factors such as variability in program adherence, availability of trained professionals, and regional disparities in healthcare infrastructure. In particular, delivering personalized nutritional counseling and group-based exercise sessions across diverse settings may require substantial human resources and logistical coordination. Policymakers and healthcare planners should consider these resource and infrastructure requirements when evaluating the scalability and sustainability of such interventions in routine practice.

Scenario analysis focusing on subgroups with demonstrated efficacy in the RCT showed that the incremental costs of scenario for targeting to APOE ε4 carriers were −1684,387 and −535,873 for targeting to the high-adherence group. However, the mechanisms underlying the preventive effects of multimodal interventions on dementia onset in APOE ε4 carriers remain unclear [17]. In addition, the scenario analysis did not account for the screening costs required to identify specific subgroups. In the future implementation of the J-MINT program, identifying APOE ε4 carriers is expected to occur in memory clinics [17]. Therefore, further research is necessary to evaluate the program's cost-effectiveness, including costs associated with screening.

Given the increasing burden of dementia and the need for sustainable healthcare systems, integrating cost-effective programs like J-MINT into national public health strategies may represent a prudent long-term investment. Policymakers should consider establishing structured, community-based prevention initiatives that incorporate multimodal interventions, especially in aging societies. Such integration could support dementia risk reduction at the population level while improving quality of life and containing long-term care costs.

### 4.1 Limitations

This study has several limitations. First, the simulation model relied on risk scores that implicitly assume direct causal relationships between included factors and dementia onset, which may lead to bias. Additionally, different cognitive measures (MMSE and CDR.) were used for transition probabilities and cost estimates, potentially causing inconsistencies and overestimation of costs. Second, intervention effects were derived from the RCT results. Real-world effectiveness may be lower due to differences in adherence and implementation conditions. Third, dementia risk reduction was estimated indirectly using the Hisayama score, which predicts rather than directly measures dementia incidence. This introduces uncertainty, and long-term follow-up is needed to validate these projections. Additionally, the Hisayama score was developed using a Japanese cohort. Therefore, its applicability to non-Japanese or more ethnically diverse populations may be limited. Finally, public long-term care costs are difficult to isolate as dementia-specific direct expenditures, which may lead to an overestimation of costs attributable to dementia. However, the estimates used in this study accounted for the distribution of care need levels by dementia severity. Given that Japan’s long-term care insurance system ties benefit limits to care need levels and calculates costs accordingly, potential overestimation due to aging-related care demands may be partially mitigated.

## 5 Conclusions

The J-MINT program demonstrated favorable cost-effectiveness, suggesting its potential as an effective strategy for dementia prevention. However, as the findings of this study were based on data from an RCT, further investigation is needed to assess the program's cost-effectiveness when implemented in real-world settings. Given the growing economic burden of dementia in Japan, incorporating multifactorial interventions such as J-MINT into public health strategies may offer significant value. Regardless, before considering wide-scale implementation, further studies are warranted to validate long-term effectiveness and clarify causal mechanisms in real-world settings. Policymakers may need to weigh the urgency of addressing dementia care challenges against the uncertainty of current evidence and consider investing in pragmatic trials or phased implementation strategies that allow for ongoing evaluation.

**Data Statement**

At present, the model code is not publicly available. However, the authors are willing to provide the annotated code upon reasonable request.

## Funding

This work was financially supported by the Japan Agency for Medical Research and Development (AMED) under Grant Number JP19de0107002 and the Research Funding for Longevity Sciences from the National Center for Geriatrics and Gerontology (23–1). The sponsors had no role in the design and conduct of the study, in the collection, analysis, and interpretation of data, in the preparation of the manuscript, or the review or approval of the manuscript.

## Consent statement

Written informed consent was obtained from all participants in the J-MINT trial.

## Disclosures

The authors have no relevant financial or non-financial interests to disclose.

## CRediT authorship contribution statement

**Naoki Takashi:** Writing – original draft, Methodology, Formal analysis. **Shosuke Ohtera:** Writing – review & editing, Supervision, Methodology, Formal analysis. **Yujiro Kuroda:** Writing – review & editing, Data curation, Conceptualization. **Hidenori Arai:** Writing – review & editing, Supervision, Conceptualization. **Takashi Sakurai:** Writing – review & editing, Supervision, Conceptualization.

## Declaration of interest

The authors declare that they have no known competing financial interests or personal relationships that could have appeared to influence the work reported in this paper.

## Appendix Supplementary materials

Image, application 1
