# Supplementary material for: Cost-effectiveness of multimodal intervention for the prevention of dementia in Japan
Source: J Prev Alzheimers Dis. 2026 Jan 1;13(2):100460. doi: 10.1016/j.tjpad.2025.100460 (PMC12869053; doi:10.1016/j.tjpad.2025.100460)
Supplement: Supplementary file 1 [file mmc1.docx]

Supplemental Table 1. Detailed cost of implementing the program

| **Type** | **item** | **Costs over the 18-month program (JPY)** | **Cost per person (JPY)** |
| --- | --- | --- | --- |
| Group physical exercise | Exercise instructor | 47,165,625 | 219,375 |
| Individual nutritional counseling | Nurse | 24,950,321 | 116,048 |
| Cognitive training development | - | 12,000,000 | 55,814 |
| Facilities | - | 7,695,000 | 35,790 |
| Device | - | 20,196,000 | 93,935 |
| **TOTAL INTERVENTION COST** | - | 113,306,865 | 520,962 |
| Health-related information | - | 94,325 | 432 |
| **TOTAL USUAL CARE COST** | - | 94,325 | 432 |
